# *PIK3CA* alterations and benefit with neratinib: analysis from the randomized, double-blind, placebo-controlled, phase III ExteNET trial

**DOI:** 10.1186/s13058-019-1115-2

**Published:** 2019-03-11

**Authors:** Stephen K. L. Chia, Miguel Martin, Frankie A. Holmes, Bent Ejlertsen, Suzette Delaloge, Beverly Moy, Hiroji Iwata, Gunter von Minckwitz, Janine Mansi, Carlos H. Barrios, Michael Gnant, Zorica Tomašević, Neelima Denduluri, Robert Šeparović, Sung-Bae Kim, Erik Hugger Jakobsen, Vernon Harvey, Nicholas Robert, John Smith, Graydon Harker, Bo Zhang, Lisa D. Eli, Yining Ye, Alshad S. Lalani, Marc Buyse, Arlene Chan

**Affiliations:** 1 British Columbia Cancer Agency, University of British Columbia, 600 West 10th Avenue, Vancouver, British Columbia V5Z4E6 Canada; 2 Instituto de Investigación Sanitaria Gregorio Marañón, Universidad Complutense de Madrid, Madrid, Spain; 3 Texas Oncology, P.A, Houston, TX USA; 4 Rigshospitalet, Copenhagen, Denmark; 5 Institut Gustave Roussy, Villejuif, France; 6 Massachusetts General Hospital Cancer Center, Boston, MA USA; 7 Aichi Cancer Center, Chikusa-ku, Nagoya, Japan; 8 Luisenkrankenhaus, German Breast Group Forschungs GmbH, Düsseldorf, Neu-isenburg, Germany; 9 Biomedical Research Centre, Guy’s Hospital, King’s College London, London, UK; 10 Pontifical Catholic University of Rio Grande do Sul School of Medicine, Porto Alegre, Brazil; 11 Department of Surgery and Comprehensive Cancer Centre, Medical University of Vienna, Vienna, Austria; 12 Daily Chemotherapy Hospital, Institute for Oncology and Radiology of Serbia, Belgrade, Serbia; 13 Virginia Cancer Specialists, Arlington, VA USA; 14 University Hospital for Tumors, Sestre Milosrdnice University Hospital Center, Zagreb, Croatia; 15 Asan Medical Center, University of Ulsan, Seoul, Korea; 16 Lillebaelt Hospital, Vejle, Denmark; 17 Auckland City Hospital, Grafton, Auckland, New Zealand; 18 McKesson Specialty Health and The US Oncology Network, The Woodlands, TX USA; 19 Compass Oncology, Portland, OR USA; 20 Utah Cancer Specialists, Salt Lake City, UT USA; 21 Puma Biotechnology, Inc., Los Angeles, CA USA; 22 International Drug Development Institute (IDDI), Louvain-la-Neuve, Belgium; 23 Breast Cancer Research Centre-WA, Perth & Curtin University, Nedlands, Australia

**Keywords:** Breast cancer, Drug targets, Neratinib, PIK3CA, Prognostic, Predictive

## Abstract

**Background:**

Neratinib is an irreversible pan-HER tyrosine kinase inhibitor that inhibits PI3K/Akt and MAPK signaling pathways after HER2 receptor activation. The ExteNET study showed that neratinib significantly improved 5-year invasive disease-free survival (iDFS) in women who completed trastuzumab-based adjuvant therapy for early breast cancer (EBC). We assessed the prognostic and predictive significance of *PIK3CA* alterations in patients in ExteNET.

**Methods:**

Participants were women aged ≥ 18 years (≥ 20 years in Japan) with stage 1–3c (modified to stage 2–3c in February 2010) operable breast cancer, who had completed (neo)adjuvant chemotherapy plus trastuzumab ≤ 2 years before randomization, with no evidence of disease recurrence or metastatic disease at study entry. Patients were randomized to oral neratinib 240 mg/day or placebo for 1 year. Formalin-fixed, paraffin-embedded primary tumor specimens underwent polymerase chain reaction (PCR) *PIK3CA* testing for two hotspot mutations in exon 9, one hot-spot mutation in exon 20, and fluorescence in situ hybridization (FISH) analysis for *PIK3CA* amplification. The primary endpoint (iDFS) was tested with log-rank test and hazard ratios (HRs) estimated using Cox proportional-hazards models.

**Results:**

Among the intent-to-treat population (*n* = 2840), tumor specimens were available for PCR testing (991 patients) and *PIK3CA* FISH (702 patients). Overall, 262 samples were *PIK3CA* altered: 201 were mutated (77%), 52 (20%) were amplified, and 9 (3%) were mutated and amplified. iDFS was non-significantly worse in placebo-treated patients with altered vs wild-type *PIK3CA* (HR 1.34; 95% CI 0.72–2.50; *P* = 0.357). Neratinib’s effect over placebo was significant in patients with *PIK3CA*-altered tumors (HR 0.41; 95% CI 0.17–0.90, *P* = 0.028) but not *PIK3CA* wild-type tumors (HR 0.72; 95% CI 0.36–1.41; *P* = 0.34). The interaction test was non-significant (*P* = 0.309).

**Conclusions:**

Although there was a greater absolute risk reduction associated with neratinib treatment of patients with *PIK3CA*-altered tumors in ExteNET, current data do not support *PIK3CA* alteration as a predictive biomarker of response to neratinib in HER2-positive EBC.

**Trial registration:**

ClinicalTrials.gov, NCT00878709. Trial registered April 9, 2009.

**Electronic supplementary material:**

The online version of this article (10.1186/s13058-019-1115-2) contains supplementary material, which is available to authorized users.

## Introduction

Randomized phase III studies have demonstrated that 12 months’ treatment with the anti-HER2 antibody trastuzumab plus adjuvant chemotherapy significantly improves clinical outcomes in women with HER2-positive early-stage breast cancer (BC) [1–3]. Despite the initial dramatic benefits with adjuvant trastuzumab, relapses are observed on longer follow-up. At the first combined analysis of the NSABP B-31 and NCCTG N9831 trials, disease-free survival (DFS) was 87.1% in trastuzumab-treated patients after a median follow-up of 2 years [4]; however, DFS was 73.7% at the 10-year follow-up [2]. In the HERA study, 2-year DFS was 85.8% in trastuzumab-treated patients (median follow-up 1 year) [5], whereas 10-year DFS after 11 years’ follow-up was estimated at 69% [1]. Longer follow-up in the BCIRG 006 trial demonstrated a similar ongoing relapse rate to earlier data in the trastuzumab-treated arms (10-year DFS 73–74.6%), particularly in patients with hormone receptor-positive and HER2-positive BC [3, 6].

Multiple mechanisms of trastuzumab resistance have been explored in early- and advanced-stage BC [7]. The phosphatidylinositol 3-kinase (PI3K)/Akt/mammalian target of rapamycin (mTOR) pathway is crucial to many physiological processes, including cell-cycle progression, apoptosis, motility, autophagy, protein and lipid synthesis, and metabolism [8]. Dysregulation of this pathway is frequently implicated in tumorigenesis and plays a key role in breast carcinoma growth and regulation [9]. Activating *PIK3CA* gene mutations occur in approximately 30–40% of BCs [10] and 22% of HER2-positive BCs [11, 12]. *PIK3CA* mutational hotspots occurring in exon 9 (E542, E545) and exon 20 (H1047) comprise approximately 70% of reported *PIK3CA* mutations in BC [10]. The significance of *PIK3CA* mutations as a biomarker of resistance to HER2-targeted therapies is inconsistent, appearing to depend on the endpoints studied and disease stage [12–21]. *PIK3CA* amplification is less frequent, with gene copy number amplification occurring in approximately 3% of BCs [10].

Neratinib, an irreversible small-molecule tyrosine kinase inhibitor of HER1, HER2, and HER4 [22], has established single-agent efficacy in patients with trastuzumab-pretreated HER2-positive metastatic BC [23, 24]. In the neoadjuvant adaptive I-SPY 2 study, neratinib plus a taxane demonstrated similar pathological complete response (pCR) rates to trastuzumab plus a taxane in patients with HER2-positive BC [25]. The ExteNET study compared 1 year of neratinib vs placebo given after standard trastuzumab-based (neo) adjuvant therapy in patients with early-stage HER2-positive BC. The primary analysis, performed after 2 years’ follow-up, showed significantly improved invasive DFS (iDFS) for neratinib vs placebo (stratified hazard ratio [HR] 0.67; 95% confidence interval [CI] 0.50–0.91; *P =* 0*.*0091) [26]. A recent update with 5-year median follow-up reported that this benefit was maintained [27]. Pre-clinically, neratinib has demonstrated potent anti-proliferative activity in HER2-amplified, *PIK3CA*-mutant tumor cell lines [28]. Furthermore, neratinib significantly inhibited tumor growth in a HER2-positive, *PIK3CA*-mutated patient-derived xenograft BC model [29]. Based on this rationale, we assessed the prognostic and predictive significance of *PIK3CA* alterations in an exploratory analysis of ExteNET.

## Patients and methods

### Study design, randomization, and masking

Details of the multicenter, randomized, double-blind, placebo-controlled phase III ExteNET study have been described previously [26]. In brief, 2840 women with histologically confirmed stage 2–3c (1–3c in original protocol) HER2-positive BC were enrolled from academic- and community-based centers in 40 countries between July 2009 and October 2011. Patients were randomized (1:1) to neratinib or placebo, given for 1 year after standard locoregional treatment, chemotherapy, and (neo)adjuvant trastuzumab. Patients, investigators, and study sponsors were masked to treatment allocation. Clinical and radiologic assessments had to be negative for recurrence or metastatic disease at study entry. (Neo)adjuvant trastuzumab was completed up to 1 year (2 years in original protocol) before randomization. All patients (intention-to-treat [ITT] population) provided written informed consent; patients in the correlative cohort signed an optional consent form relating to primary tumor collection for exploratory biomarker analyses.

Three different sponsors were involved over the course of the study, resulting in three global amendments to the study design [26]; the correlative study was developed with amendment 3 and with the initial study sponsor. The Independent Data Monitoring Committee (IDMC) remained consistent throughout the study to preserve blinding integrity; the infrastructure for study conduct and monitoring remained in place to preserve operational consistency. The IDMC reviewed the data at least twice yearly. Additional study design details are described in Additional file 1.

The aim of the present analysis was to assess the prognostic and predictive significance of *PIK3CA* alterations in ExteNET.

### Procedures

Patients were randomized to neratinib (Puma Biotechnology, Los Angeles, CA, USA) 240 mg orally once daily continuously or matching placebo for 1 year. Concurrent adjuvant endocrine therapy for women with locally determined hormone receptor-positive disease was permitted and recommended.

Tumor blocks or freshly cut, unstained sections mounted on positively charged slides were sent to a central certified laboratory. *PIK3CA* gene testing by reverse transcriptase polymerase chain reaction (RT-PCR) and/or fluorescence in situ hybridization (FISH) was performed as materials were received from August 2009 through June 2011. *PIK3CA* FISH was used to quantify *PIK3CA* gene copy number in relation to the number of copies of chromosome 3 using Vysis LSI *PIK3CA* Spectrum Green and Vysis CEP 3 Spectrum Orange Probes (Abbott Molecular, Abbott Park, IL, USA). Slides were deparaffinized and pretreated using Vysis Paraffin Pretreatment Reagent Kit II (Abbott Molecular). Probe mixes were hybridized at 37 °C for 17 h. Hybridized slides were counterstained with DAPI II. A minimum of 50 nuclei of invasive tumor cells were scored by a board-certified pathologist using a LEICA microscope at × 100 objective. Amplification status was determined from the ratio of *PIK3CA* to chromosome 3 centromere probe (CEP3) signals. A ratio ≥ 2.2 was considered FISH positive and < 2.2 was considered normal. The pathologist was blinded to the clinical data, randomized arm, and outcome throughout the pre-analytical and analytical process.

The proportion of tumor nuclei present was determined by a board-certified pathologist. Macro-dissection for tumor enrichment was performed if the tumor burden was < 20%. Genomic DNA was extracted from formalin-fixed, paraffin-embedded (FFPE) tumor slides (QiaAmp DNA FFPE tissue kit, Qiagen, Valencia, CA, USA). Genomic DNA (5 ng/μL) was tested for *PIK3CA* mutations (DxS PI3K Mutation Test, DxS, Manchester, UK) using RT-PCR to qualitatively detect E542K, E545D/E545K, and H1047R at a minimum detection level of 1% H1047R, E542K, and E545K or 2% E545D relative to the background. E545D and E545K mutations were detected in a single reaction; both were detectable but not distinguishable. PCR was performed using an ABI 7900HT machine and analyzed using SDS version 2.2.2 software (Applied Biosystems, Foster City, CA, USA).

### Statistical analysis

Biomarker results were based on 5-year iDFS data. iDFS was the time from randomization to the first occurrence of invasive ipsilateral BC recurrence, invasive contralateral BC, local/regional invasive recurrence, distant recurrence, or death from any cause. Patients without DFS events were censored at the date of last physical examination. Exploratory *PIK3CA* biomarker analyses were performed in the correlative cohort, which included patients with samples tested for *PIK3CA* mutation and/or *PIK3CA* amplification.

Data from the placebo arm were used for the prognostic evaluation of *PIK3CA* alterations. The *PIK3CA*-altered group comprised patients with mutated and/or amplified *PIK3CA*. The *PIK3CA* wild-type group comprised of patients with no measurable *PIK3CA* mutation or amplification. Kaplan-Meier methods were used to estimate annual event-free survival rates. Log-rank tests were used to compare 5-year iDFS between the *PIK3CA*-altered and wild-type groups in the placebo arm. HRs were estimated using the Cox regression model.

For sensitivity analysis, a multivariate Cox model was fitted to estimate the treatment effect within the subgroups, adjusting for baseline prognostic factors including age, baseline Eastern Cooperative Oncology Group performance status, race, region, menopausal status, nodal status, hormone receptor status, histological grade, radiotherapy, prior trastuzumab, time from last trastuzumab, prior surgery, and prior neoadjuvant therapy. In addition, the interaction between *PIK3CA* status (altered vs wild-type) and treatment (neratinib vs placebo) was tested using a Cox regression model. All analyses were exploratory, with no adjustment for multiplicity, and were performed using SAS statistical software (version 9.3; SAS Institute Inc., Cary, NC, USA).

## Results

### Participants

The ITT population included 2840 women randomly assigned to the study treatment (neratinib, *n* = 1420; placebo, *n* = 1420) between July 9, 2009, and October 24, 2011; 1201 patients (42.3%) consented to the inclusion in the correlative cohort and had sufficient tumor material for biomarker testing using at least 1 of the testing methods (neratinib, *n* = 593; placebo, *n* = 608) (Additional file 1: Figure S1). Baseline demographics and disease characteristics were well balanced between correlative cohort treatment arms and were similar to the ITT population (Table 1). In the ITT population, 5-year iDFS was 90.2% (95% CI 88.3–91.8%) for neratinib and 87.7% (95% CI 85.7–89.4%) for placebo, giving an absolute benefit of 2.5% for neratinib vs placebo (HR 0.73; 95% CI 0.57–0.92; *P =* 0*.*008) [24]. In the correlative cohort, 5-year iDFS was 91.0% (95% CI 88.1–93.2%) for neratinib vs 87.8% (95% CI 84.8–90.3%) for placebo (HR 0.67; 95% CI 0.45–0.96; *P =* 0*.*032). Follow-up continues until the final analysis of the overall survival (OS) is reported.

**Table 1:** Baseline demographics and disease characteristics for the ITT population and the correlative cohort

Characteristic	ITT population (*n* = 2840)		Correlative cohort (*n* = 1201)	
	Neratinib (*n* = 1420)	Placebo (*n* = 1420)	Neratinib (*n* = 593)	Placebo (*n* = 608)
Median age, years (range)	52 (25–83)	52 (23–82)	53 (26–83)	53 (26–81)
Menopausal status at diagnosis, *n* (%)				
Premenopausal	663 (46.7)	664 (46.8)	277 (46.7)	267 (43.9)
Postmenopausal	757 (53.3)	756 (53.2)	316 (53.3)	341 (56.1)
Prior trastuzumab, *n* (%)				
Concurrent to chemotherapy	884 (62.3)	886 (62.4)	374 (63.1)	400 (65.8)
Sequential after chemotherapy	536 (37.7)	534 (37.6)	219 (36.9)	208 (34.2)
Median time since trastuzumab, months (range)	4.4 (0.2–30.9)	4.6 (0.3–40.6)	4.9 (0.3–24.0)	6.5 (0.4–24.2)
Median time from diagnosis to randomization (range), months	21.82 (7.69–73.69)	22.29 (7.82–103.03)	22.44 (7.79–73.69)	23.64 (7.82–66.96)
Nodal status, *n* (%)				
Negative	335 (23.6)	336 (23.7)	160 (27.0)	177 (29.1)
1–3 positive nodes	664 (46.8)	664 (46.8)	265 (44.7)	266 (43.8)
≥ 4 positive nodes	421 (29.6)	420 (29.6)	168 (28.3)	165 (27.1)
Hormone receptor status, *n* (%)				
Positive	816 (57.5)	815 (57.4)	339 (57.2)	361 (59.4)
Negative	604 (42.5)	605 (42.6)	254 (42.8)	247 (40.6)

### Distribution of *PIK3CA* alterations

**Fig. 1:**
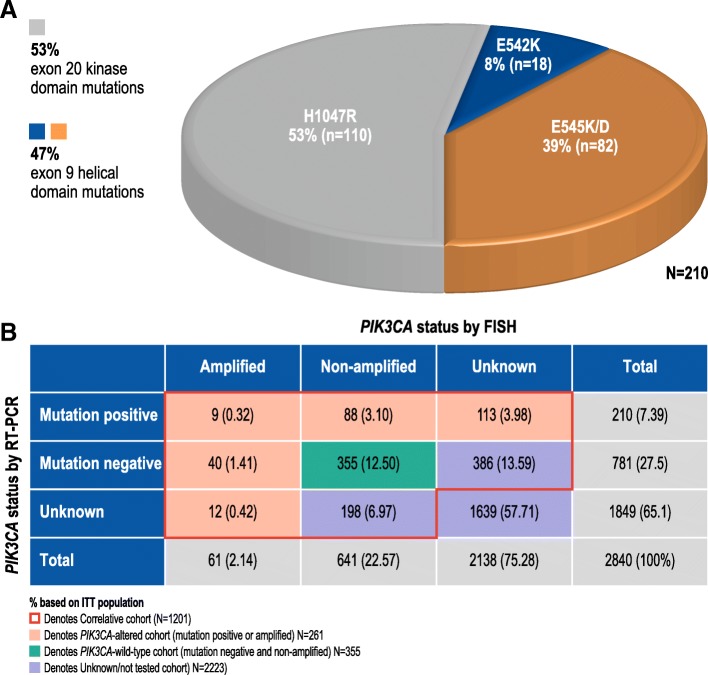
*PIK3CA* mutations and *PIK3CA* amplifications in the correlative cohort. **a** Distribution of hotspot variants in *PIK3CA* mutation-positive subgroups. **b** Distribution of *PIK3CA* amplification or mutation status in the correlative cohort

Tumor samples from 991 correlative cohort patients successfully underwent RT-PCR for the assessment of exon 9 E542K and E545K/D mutations and exon 20 H1047R mutation. A total of 210 tumors (21.2%) had 1 of these mutations, consistent with previous reports [10]: 110 mutations (53%) in exon 20 (H1047R), 18 (8%) in exon 9 E542K, and 82 (39%) in exon 9 E545K/D (Fig. 1a). *PIK3CA* mutations were detected in 129 of 576 (22.4%) hormone receptor-positive patients and in 81 of 415 (19.5%) hormone receptor-negative patients. Furthermore, 702 samples underwent FISH analyses, 61 (8.7%) of which were deemed *PIK3CA* amplified (*PIK3CA*:CEP3 ≥ 2.2). Nine samples were both mutation-positive and *PIK3CA* amplified; however, over half of the mutation-positive samples had unknown *PIK3CA* amplification status by FISH (Fig. 1b). *PIK3CA* gene amplification was detected in 28 of 408 patients (6.9%) with hormone receptor-positive disease and 33 of 294 (11.2%) with hormone receptor-negative disease.

### Prognostic effects

The prognostic effect of *PIK3CA* alterations was assessed in 312 patients in the placebo arm of the correlative cohort who had sufficient tumor material for the testing of *PIK3CA* mutations and *PIK3CA* amplification. There was no significant association in iDFS observed in patients harboring *PIK3CA* alteration (HR 1.34; 95% CI 0.72–2.50) (*P =* 0*.*357). Estimated 5-year iDFS was 88.8% in the *PIK3CA* wild-type group vs 84.5% in the *PIK3CA*-altered group (Additional file 1: Figure S2).

### Treatment effects

**Fig. 2:**
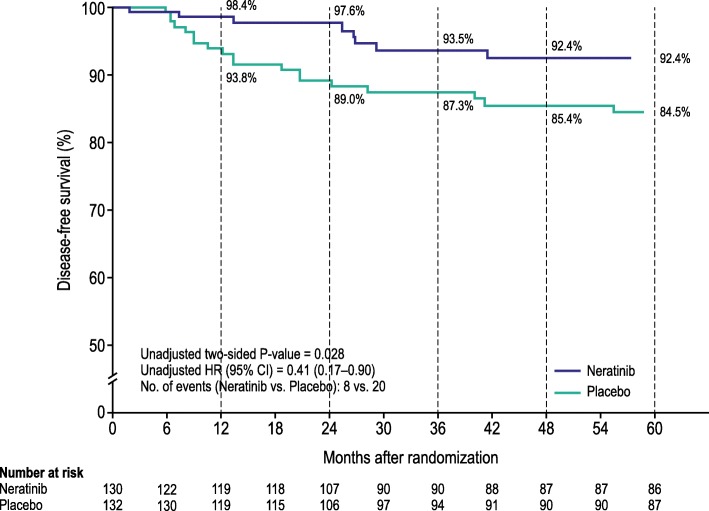
Kaplan-Meier plot of 5-year invasive disease-free survival for patients with *PIK3CA*-altered tumors (correlative cohort)

The predictive effect of *PIK3CA* alterations on response to neratinib was assessed by comparing 5-year iDFS rates for neratinib-treated and placebo-treated *PIK3CA*-altered patients. In *PIK3CA*-altered patients, 5-year iDFS was 92.4% for neratinib vs 84.5% for placebo (HR 0.41, 95% CI 0.17–0.90; *P =* 0*.*028) (Table 2; Fig. 2). In *PIK3CA* wild-type patients (mutation-negative and non-amplified), the treatment effect of neratinib was less pronounced (5-year iDFS 90.6% vs 88.8%; HR 0.72, 95% CI 0.36–1.41; *P =* 0*.*340) (Additional file 1: Figure S3). The interaction test for the *PIK3CA* subgroups was not statistically significant (*P =* 0*.*309).

**Table 2:** Five-year iDFS events in patients treated with neratinib and placebo relative to *PIK3CA* status

Population	Neratinib		Placebo			
	*n*	iDFS events, *n*	*n*	iDFS events, *n*	HR (95% CI)	Two-sided *P* value^a^
ITT	1420	116	1420	163	0.73 (0.57–0.92)^b^	0.008^b^
Correlative cohort	593	45	608	70	0.67 (0.45–0.96)	0.032
*PIK3CA* mutation positive	104	7	106	17	0.43 (0.17–1.01)	0.056
*PIK3CA* mutation negative	385	27	396	42	0.66 (0.40–1.06)	0.089
*PIK3CA* amplified	33	1	28	4	0.20 (0.01–1.33)	0.106
*PIK3CA* non-amplified	316	29	325	36	0.85 (0.52–1.39)	0.521
*PIK3CA* altered^c^	130	8	132	20	0.41 (0.17–0.90)	0.028

A total of nine patients (seven patients in the neratinib arm and two patients in the placebo arm) had tumors harboring both a mutation and amplification

^a^Log-rank test

^b^Stratified analysis

^c^Tumors harboring either a *PIK3CA* mutation or *PIK3CA* amplification

**Fig. 3:**
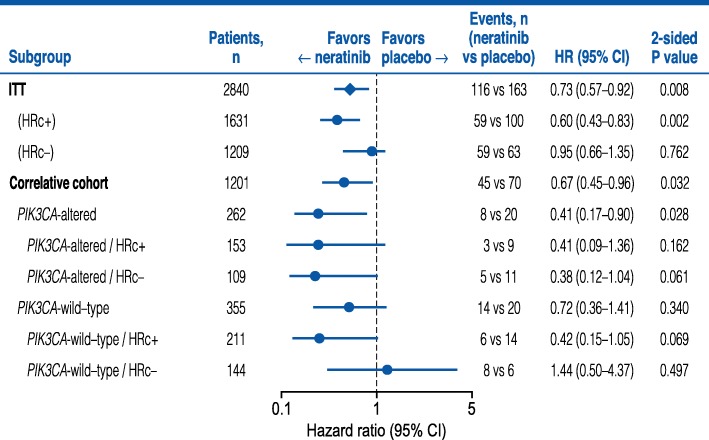
Forest plot of neratinib vs placebo in PIK3CA-altered and hormone receptor subgroups. HRc, hormone receptor

A multivariate analysis adjusting for clinical prognostic covariates revealed a statistically significant benefit for neratinib vs placebo in patients with *PIK3CA*-altered tumors (HR 0.40, 95% CI 0.15–0.92; *P =* 0*.*041) (Additional file 1: Table S1). The proportion of patients with hormone receptor-positive tumors was similar in *PIK3CA*-altered (58.4%) and wild-type subgroups (59.4%). The observed benefit for neratinib in patients with *PIK3CA*-altered tumors was independent of hormone receptor status (Fig. 3).

## Discussion

Studies have demonstrated that *PIK3CA* mutations and amplifications contribute to the hyperactivation of the PI3K/Akt pathways in breast and other human cancers [30–36]. The contribution of *PIK3CA* gene amplifications in breast cancer tumorigenesis is not well studied, and gene amplifications are relatively infrequent in breast cancers compared to somatic mutations. In the present analysis of data from the ExteNET study, we evaluated *PIK3CA* amplifications and somatic mutations to obtain a comprehensive assessment of the prognostic and predictive significance of *PIK3CA* alterations in patients with early-stage HER2-positive breast cancer.

The ExteNET study demonstrated that 1 year of neratinib, given after standard trastuzumab-based adjuvant therapy, significantly improved iDFS in women with early-stage HER2-positive BC, with a 27% relative reduction in the risk of an iDFS event (stratified HR 0.73, 95% CI 0.57–0.92; *P =* 0*.*008), corresponding to an absolute improvement of 2.5% vs placebo [27]. In patients with hormone receptor-positive disease, most of whom received concurrent adjuvant hormonal therapy with neratinib/placebo, the magnitude of benefit was more pronounced (HR 0.60, 95% CI 0.43–0.83; *P =* 0*.*002) with an absolute improvement of 4.4%. The present correlative study attempted to further refine the HER2-positive patient population that may benefit based on *PIK3CA* alterations within their primary tumor. Patients with *PIK3CA*-altered tumors derived numerically greater benefit from neratinib than placebo: 5-year iDFS was numerically better with neratinib vs placebo, equating to an absolute improvement of 7.9% in favor of neratinib. Furthermore, neratinib’s benefit appeared similar in patients with hormone receptor-positive and hormone receptor-negative *PIK3CA*-altered tumors; however, as the interaction test between *PIK3CA* status and neratinib benefit was not statistically significant, we cannot definitively conclude that *PIK3CA* status is predictive in nature for selection of neratinib therapy.

*PIK3CA* alterations are among the most common genomic aberrations seen in patients with HER2-positive BC; data suggest that *PIK3CA* is altered in 36% of patients [10], with mutations in 30.7%, amplifications in 3.1%, and multiple alterations in 2.1% of cases [37]. Similar rates of *PIK3CA* alterations (35.9%) were reported in another cohort study, primarily comprising advanced-stage BC [38]. In their recent large pooled analysis of outcomes in patients with early-stage HER2-positive BC, Zavardas and colleagues reported an overall *PIK3CA* mutation rate of 22% [11], similar to the 21% mutation rate we report. We observed a higher amplification rate (8.7%) than previously reported [10], which may be related to discrepancies in gene amplification measurement methods (FISH vs copy number alterations by next-generation sequencing). Within The Cancer Genome Atlas dataset, 70% of *PIK3CA* mutations were at hotspots E542, E545, or H1047 [10]. Although available techniques at the time of measurement (2009–2011) only allowed us to profile two hotspot mutations in exon 9 (E542K, E545K/D) and one hotspot mutation in exon 20 (H1047R), these appear to account for over two thirds of the reported mutations across the *PIK3CA* gene [10].

The prognostic and predictive significance of *PIK3CA* alterations, particularly mutations, have been extensively studied in BC. In metastatic BC, *PIK3CA* mutation status showed the greatest prognostic effect of those analyzed in the CLEOPATRA study [19]. *PIK3CA* status was an independent poor prognostic factor, with 8.6 months’ median progression-free survival (PFS) for mutated *PIK3CA* vs 13.8 months for wild-type *PIK3CA* in the control arm, and 12.5 months for mutated *PIK3CA* vs 21.8 months for wild-type *PIK3CA* in the pertuzumab arm. In the EMILIA study, *PIK3CA* mutations were associated with shorter PFS and OS in capecitabine plus lapatinib-treated patients, but interestingly not in T-DM1-treated patients [20].

The prognostic effect of *PIK3CA* mutations is less clear in early-stage disease, with seeming variability between short- and long-term outcome and across different clinical trials [11–13, 17, 18]. In our correlative analysis of ExteNET, patients with *PIK3CA*-mutant tumors did not have a statistically significant difference in survival compared with patients with PIK3CA wild-type tumors.

The presence of *PIK3CA* alterations and a statistically significant differential benefit from HER2-directed therapies has not been demonstrated in prior studies. In the pooled analysis of five neoadjuvant studies, the interaction test was not significant for *PIK3CA* status and benefit from combination vs single-agent HER2-directed treatments [12]. Results from FinHER [17] and NSABP B-31 [18] also failed to demonstrate a significant interaction between *PIK3CA* mutation and the degree of benefit from adjuvant trastuzumab relative to control. Although we observed a greater absolute improvement in iDFS for neratinib in *PIK3CA-*altered vs wild-type tumors in the present study, the interaction test was not statistically significant (*P =* 0*.*1842), and thus, we cannot conclude that *PIK3CA* status is predictive of benefit for neratinib following 1 year of trastuzumab in patients with early-stage HER2-positive BC.

One limitation of this study is that only three *PIK3CA* mutation hotspots were assessed, although the PCR methodology used provided good sensitivity for detecting mutations even at low allele frequencies. Today, however, a more comprehensive, whole-exome, or next-generation sequencing might be performed to detect the full spectrum of genomic aberrations across the *PIK3CA* gene. It is unclear if other known *PIK3CA* variants would trend toward a similar benefit for neratinib as seen for the E542K, E545K/D, and H1047R variants. A second limitation is that validated guidelines for a definition for PI3K amplification to be associated with biological relevance have not yet been established. Since guidelines for *PIK3CA* amplification have not been established, we used the same cutoff that was defined for HER2 FISH amplification by ASCO/CAP at the time of sample testing. Another limitation is that FFPE tumor tissue was only collected from 42% of the ITT population, and only 41% of these samples were tested for both *PIK3CA* amplification and mutation. These tissues were archival and the primary resected breast specimen, and thus reflect the state of the tumor prior to receiving any systemic therapy. Although the study results were essentially unchanged after adjusting for important prognostic factors, the possibility of bias cannot be excluded. A larger sample size would have improved the likelihood of demonstrating a statistically significant interaction test, if one truly exists. A final limitation was the fact that patients with early relapse during adjuvant trastuzumab-based therapy were excluded from ExteNET as patients had to be clinically disease-free at the study entry, with a median time from diagnosis to randomization of 22–24 months. Thus, the results and hypotheses associated with our study may not be relevant in those tumors with primary resistance to adjuvant trastuzumab.

## Conclusions

The use of biomarkers to identify patient populations likely to benefit from targeted therapies is a key challenge in the treatment of patients with breast cancer. The clinical significance of *PIK3CA* mutation as a biomarker of resistance to HER2-targeted therapies has not been clearly established. This exploratory correlative study reports a trend toward the absolute risk reduction for neratinib treatment being greater in patients with *PIK3CA*-altered vs wild-type HER2-positive BC following standard adjuvant chemotherapy and trastuzumab; however, this observation was not statistically significant. Therefore, these results do not support *PIK3CA* alterations as a predictive biomarker for benefit from neratinib treatment. Consideration of *PIK3CA* status as a definitive factor for the use of neratinib in the adjuvant setting would require a prospective clinical study with sufficient power to assess predictive significance for the biomarker.

## Additional file

Additional file 1: Supplementary methods. Table S1. Multivariate analyses adjusting for clinical prognostic covariates. Figure S1. ExteNET: CONSORT diagram of the correlative cohort. Figure S2. Kaplan-Meier plots of 5-year invasive disease-free survival for *PIK3CA*-altered vs wild-type tumors in the placebo arm of the correlative cohort, for assessment of prognostic effect. Figure S3. Kaplan-Meier plot of 5-year invasive disease-free survival for PIK3CA wild-type patients in the correlative cohort. (DOCX 404 kb)

## Abbreviations

BC: Breast cancer
CEP3: Chromosome 3 centromere probe
CI: Confidence interval
DFS: Disease-free survival
FFPE: Formalin-fixed, paraffin-embedded
FISH: Fluorescence in situ hybridization
HR: Hazards ratio
IDMC: Independent Data Monitoring Committee
ITT: Intention-to-treat
mTOR: Mammalian target of rapamycin
OS: Overall survival
PI3K: Phosphatidylinositol 3-kinase
RT-PCR: Reverse transcriptase polymerase chain reaction
