# Supplementary material for: PIK3CA alterations and benefit with neratinib: analysis from the randomized, double-blind, placebo-controlled, phase III ExteNET trial
Source: Breast Cancer Res. 2019 Mar 11;21:39. doi: 10.1186/s13058-019-1115-2 (PMC6417207; doi:10.1186/s13058-019-1115-2)

# Supplementary Online Content

# Supplementary Methods

## Study design

On Oct 14, 2011, two key changes were made by the sponsor at the time (amendment 9): cessation of enrolment and shortening of follow-up from 5 years to 2 years from randomisation. This decision was not made as a result of predefined futility boundaries having been met, any interim assessment of efficacy, or because of safety concerns. The study continued with this design until January 2014, when a global amendment by the current sponsor restored the primary endpoint of invasive disease-free survival to the intention-to-treat population, as defined in the original protocol, but with the primary analysis being conducted in all patients at 2 years of follow-up. Thus, all patients in the intention-to-treat population who had undergone protocol-specified treatment and follow-up to 24 months were included in this primary analysis report. Data collection for disease events and deaths from 2 years to 5 years after randomisation was resumed, with ongoing long-term survival follow-up for consenting patients. Treatment assignment remained masked before this primary analysis and the sponsor remained masked to treatment allocation for overall survival events.

## Randomization and Masking

Patients were randomly assigned (1:1) to receive neratinib or a visually identical matching placebo. The randomization sequence was generated with permuted blocks and stratified according to locally determined hormone receptor status (hormone receptor-positive, ie either oestrogen or progesterone receptor-positive or both vs hormone receptor-negative ie oestrogen and progesterone receptor-negative), nodal status (0, 1–3, or ≥4), and trastuzumab adjuvant regimen (sequential vs concurrent with chemotherapy), then implemented centrally via an interactive voice and web-response system. Once the patient was allocated to a treatment group, site personnel were provided with a subject randomization number and package number.

The study was conducted in a double-blind manner until the primary analysis (July 2014), at which time treatment allocation was unmasked to the Puma Biotechnology team responsible for the analysis. After the primary analysis, the funder established a firewall so that the team responsible for the collection of invasive disease-free survival and survival data remained masked to treatment allocation, thereby maintaining the integrity of the analyses.

**Table S1.** Multivariate analyses adjusting for clinical prognostic covariates

| **Population** | **Neratinib** | | **Placebo** | | **HR (95% CI)** | |
| --- | --- | --- | --- | --- | --- | --- |
|  | ***n*** | **iDFS events, *n*** | ***n*** | **iDFS events, *n*** | **Unadjusted** | **Adjusted** |
| Correlative cohort | 593 | 45 | 608 | 70 | 0.67 (0.45–0.96) | 0.65 (0.44–0.95) |
| *PIK3CA* altered | 130 | 8 | 132 | 20 | 0.41 (0.17–0.90) | 0.40 (0.15–0.92) |
| *PIK3CA* wild type | 175 | 14 | 180 | 20 | 0.72 (0.36–1.41) | 0.68 (0.33–1.38) |
| Unknown/not tested | 1115 | 94 | 1108 | 123 | 0.79 (0.60–1.03) | 0.79 (0.60–1.03) |

Multivariate Cox regression full model adjusting for all critical prognostic co-variables including: age; baseline Eastern Cooperative Oncology Group performance status; race; region; menopausal status; nodal status; hormone-receptor status.

**Figure S1.** ExteNET: CONSORT diagram of the correlative cohort.

**
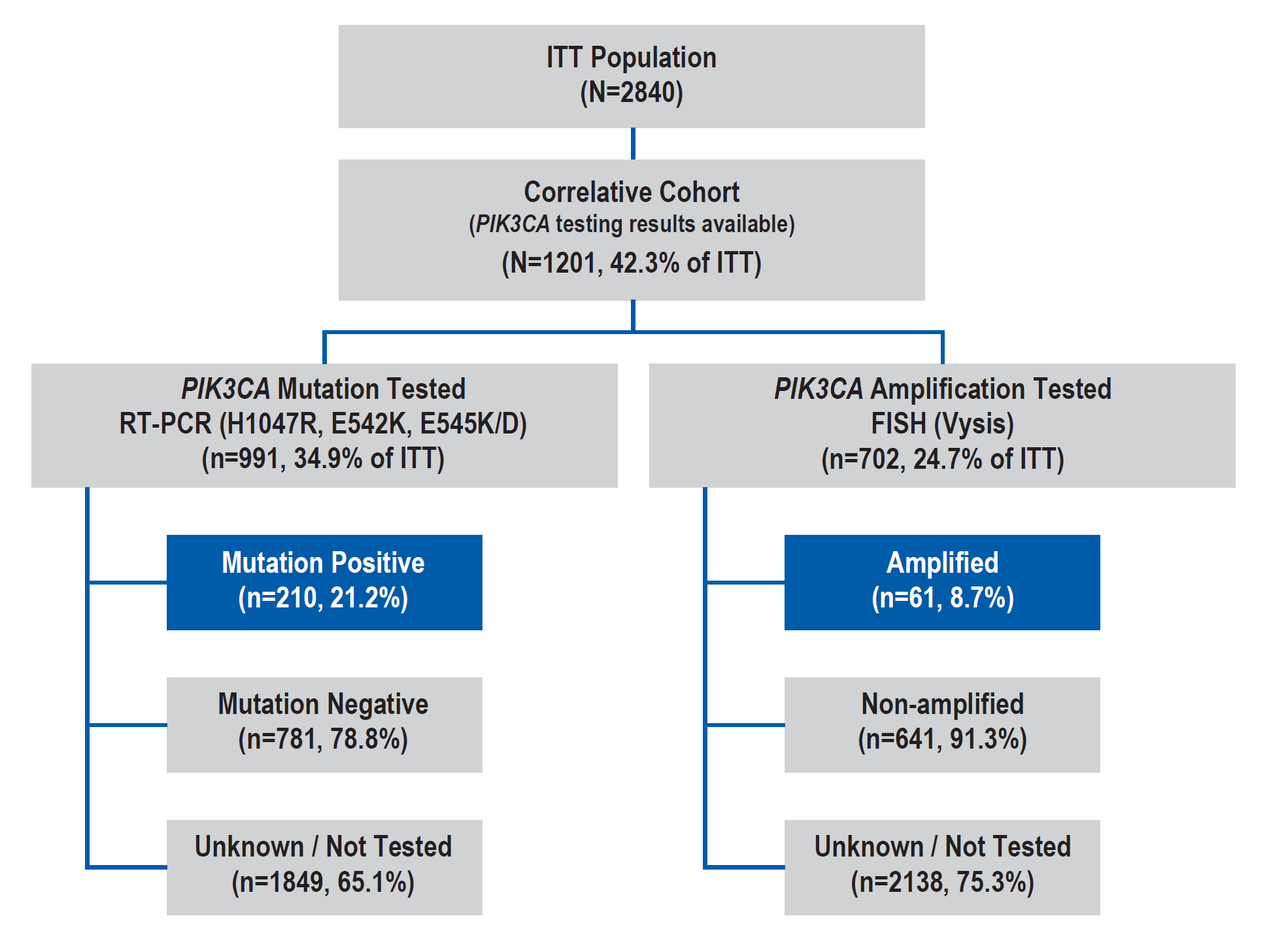
**

**Figure S2.** Kaplan-Meier plots of 5-year invasive disease-free survival for *PIK3CA*-altered vs wild-type tumors in the placebo arm of the correlative cohort, for assessment of prognostic effect. (A) *PIK3CA*-altered (mutant or amplified) versus wild-type tumors for patients in the placebo treatment group; (B) *PIK3CA*-mutant versus wild-type for tumors in the placebo treatment group.


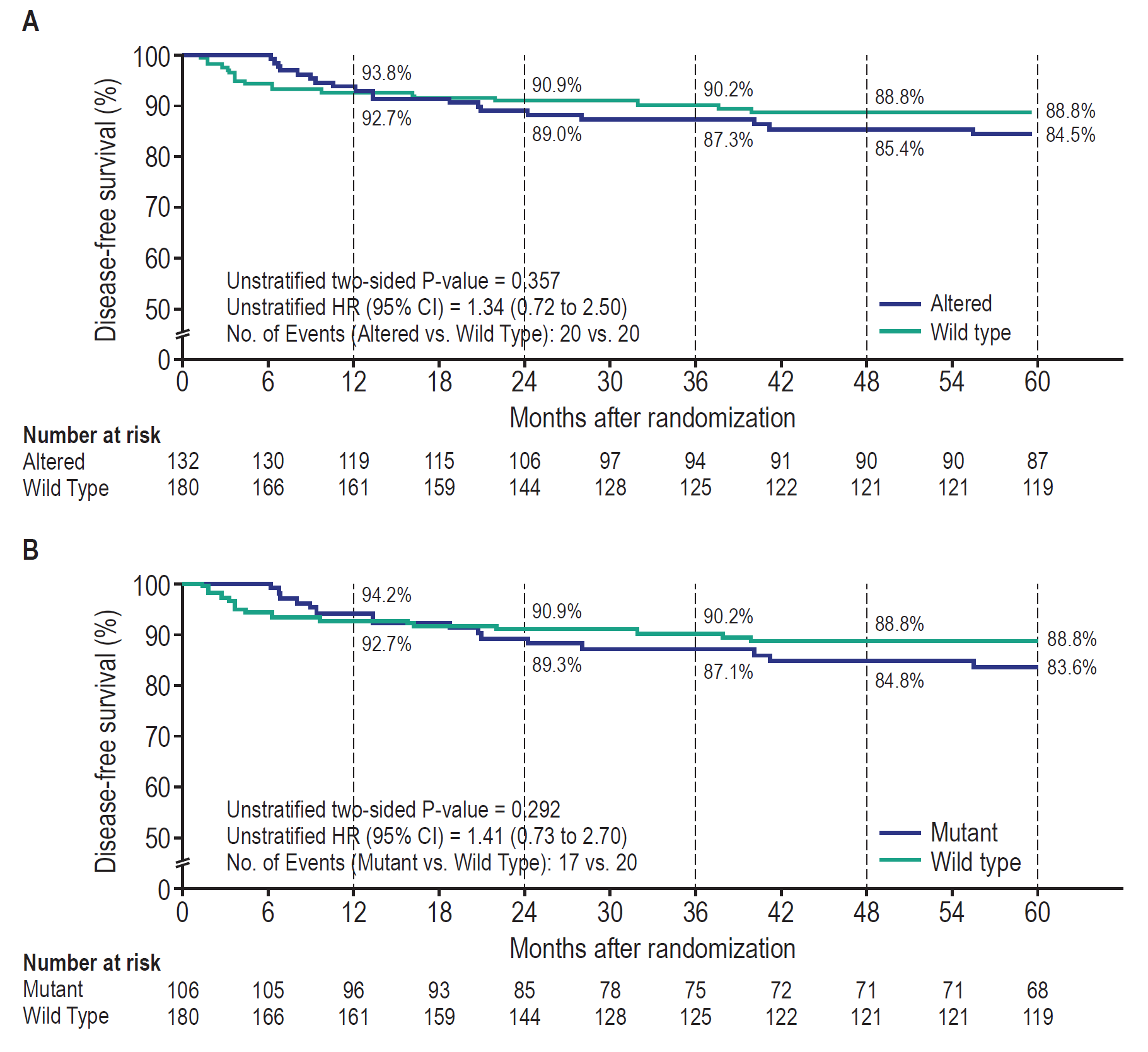


**Figure S3.** Kaplan-Meier plot of 5-year invasive disease-free survival for *PIK3CA* wild-type patients in the correlative cohort.


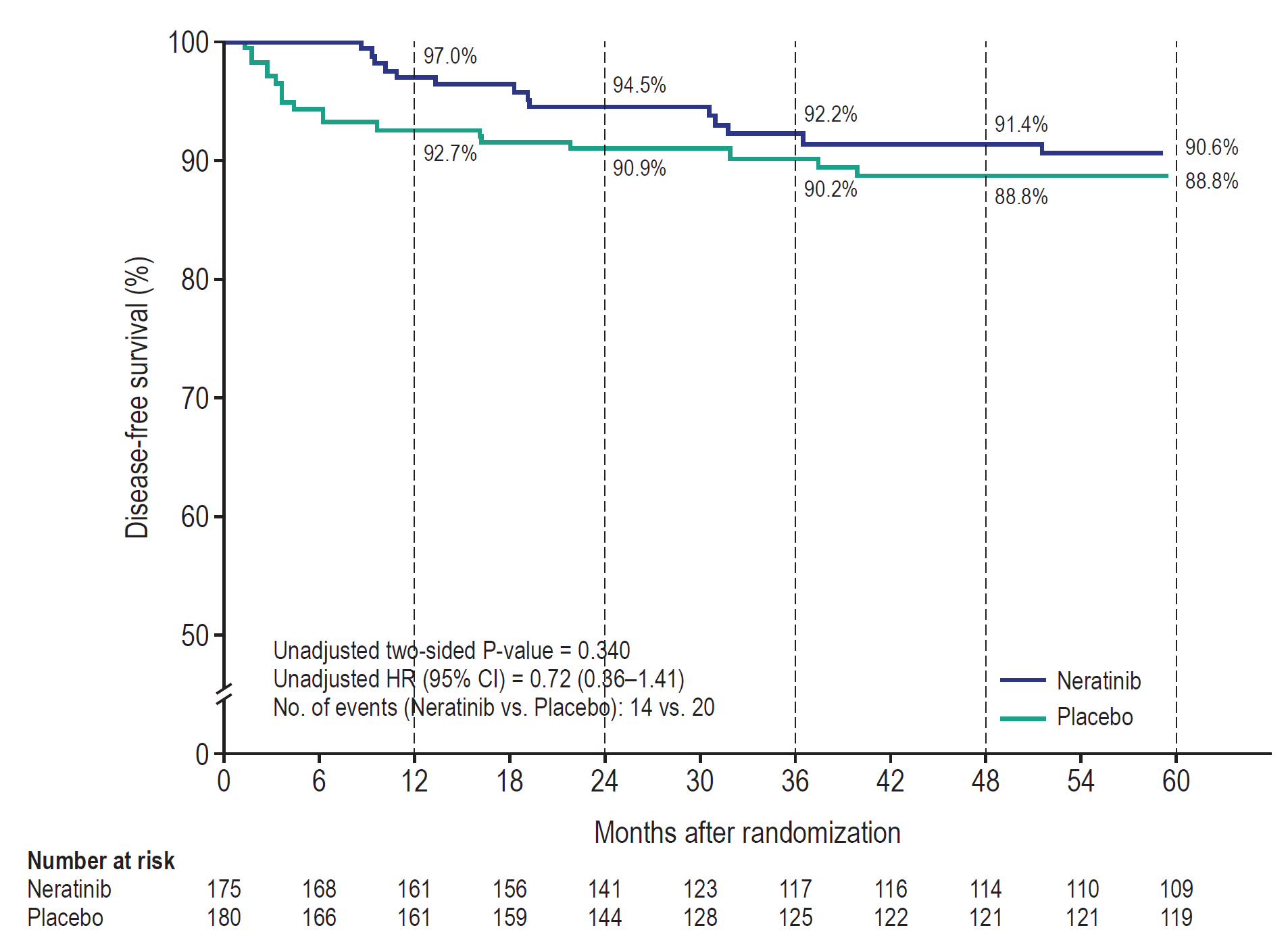

Supplement: Supplementary file 1 — Supplementary methods. Table S1. Multivariate analyses adjusting for clinical prognostic covariates. Figure S1. ExteNET: CONSORT diagram of the correlative cohort. Figure S2. Kaplan-Meier plots of 5-year invasive disease-free survival for PIK3CA-altered vs wild-type tumors in the placebo arm of the correlative cohort, for assessment of prognostic effect. Figure S3. Kaplan-Meier plot of 5-year invasive disease-free survival for PIK3CA wild-type patients in the correlative cohort. (DOCX 404 kb) [file 13058_2019_1115_MOESM1_ESM.docx]
